# Investigating the Anrep Effect in Hypertrophic Obstructive Cardiomyopathy With Invasive Pressure-Volume Analysis

**DOI:** 10.1016/j.jacadv.2025.101728

**Published:** 2025-04-25

**Authors:** Jan-Christian Reil, Vasco Sequeira, Gert-Hinrich Reil, Smita Scholtz, Volker Rudolph, Christoph Maack, Patrick Serruys, Paul Steendijk

**Affiliations:** a Klinik für allgemeine und interventionelle Kardiologie, Herz-und Diabetes Zentrum Nordrhein-Westphalen, Bad Oeynhausen, Germany; b Comprehensive Heart Failure Center, University Clinic Würzburg, Würzburg, Germany; c Universitätsklinik für Innere Medizin–Kardiologie, Klinikum Oldenburg, Oldenburg, Germany; d CORRIB Research Centre for Advanced Imaging and Core Laboratory, University of Galway, Galway, Ireland; e Department of Cardiology, Leiden University Medical Center, Leiden, the Netherlands

**Keywords:** hypertrophic obstructive cardiomyopathy, Anrep effect, afterload, Anrep triad, septal ablation

## Abstract

**Background:**

The Anrep effect, an adaptation enhancing left ventricular (LV) contractility in response to raised afterload, is hypothesized to influence hypertrophic cardiomyopathy pathophysiology.

**Objectives:**

This study investigated the activation and reversibility of the Anrep effect in obstructive hypertrophic cardiomyopathy (HOCM) patients undergoing percutaneous transluminal septal myocardial ablation (PTSMA) to relieve LV outflow tract obstruction.

**Methods:**

Invasive pressure-volume (PV) analysis was performed on 14 HOCM patients before and after PTSMA. The “Anrep Triad,” defined by elevated afterload (higher LV end-systolic pressure and effective arterial elastance), augmented contractility (higher end-systolic elastance and maximum LV pressure rise [dP/dt_max_]), and prolonged systolic duration (dTes), was assessed via direct hemodynamic comparison preprocedure and postprocedure. Stroke work (SW), potential energy, and total PV area (PVA) quantified mechanical work and efficiency (SW/PVA).

**Results:**

Postprocedure reversal of the Anrep effect was confirmed (pre- vs post-PTSMA), with reductions in afterload (LV end-systolic pressure: 180 vs 138 mm Hg, *P* = 0.0001; effective arterial elastance: 2.5 vs 1.9 mm Hg/mL, *P* = 0.002), contractility (end-systolic elastance: 2.0 vs 1.5 mm Hg/mL, *P* = 0.0001; dP/dt_max:_ 1,775 vs 1,560 mm Hg/s, *P* = 0.017), and systolic duration (dTes: 371 vs 327 ms, *P* = 0.002). Preprocedure, HOCM patients exhibited higher mechanical workload (SW: 8,161 vs 7,495 mm Hg·mL, *P* = 0.004; potential energy: 7,837 vs 4,915 mm Hg·mL, *P* = 0.002; PVA: 16,135 vs 11,742 mm Hg·mL, *P* = 0.0002) and lower efficiency (SW/PVA: 50% vs 59%, *P* = 0.03).

**Conclusions:**

The Anrep effect is an energy-demanding compensatory mechanism that maintains stroke volume under elevated afterload by increasing contractility and prolonging systole. This study confirms its chronic activation in HOCM and its immediate reversal post-PTSMA.

Hypertrophic cardiomyopathy (HCM) is the most common inherited heart disease, characterized by left ventricular (LV) hypertrophy. Diagnosis is based on an end-diastolic LV wall thickness of at least 15 mm, or 13 to 14 mm in individuals with a family history of HCM or a positive genetic test.^1^^,^^2^ Despite established genetic links to mutations in various sarcomeric genes, nearly 65% of symptomatic patients lack these common pathogenic variants.^3^ Key features of HCM include hyperdynamic systolic function, diastolic dysfunction, and significant hypertrophy of the interventricular septum, often accompanied by mitral valve abnormalities.^4^ These frequently lead to LV outflow tract obstruction (LVOTO) in up to two-thirds of patients, primarily due to systolic anterior motion (SAM) of the mitral valve.^1^^,^^2^ LVOTO increases afterload, raises LV end-systolic pressure (LVESP), and exacerbates hypercontractility, myocardial oxygen consumption, diastolic dysfunction, secondary mitral regurgitation, and the risk of heart failure and sudden cardiac death.^5^, ^6^, ^7^ Clinically, LVOTO is associated with symptoms like exercise intolerance, dyspnea, fatigue, and syncope.^2^^,^^8^ Treatments such as septal myectomy or percutaneous transluminal septal myocardial ablation (PTSMA) are the primary methods to alleviate LVOTO and reduce afterload.^8^ Patients with HCM and LVOTO are classified as having hypertrophic obstructive cardiomyopathy (HOCM). Our previous research hinted at a link between chronic high afterload from LVOTO and hyperdynamic contraction, potentially due to the prolonged activation of the Anrep effect in HOCM.^9^

Historically viewed as a transient, nonpathological adaptation, the Anrep effect augments LV contractility in response to the sudden rise in afterload.^10^^,^^11^ Although its physiological importance is well-documented, its clinical relevance has largely gone unrecognized. Using the working heart approach in isolated healthy mouse hearts, we previously characterized the Anrep effect's hemodynamic signature during acute afterload elevations at constant preload and heart rate.^12^ This signature is summarized by a triad of responses: 1) elevated afterload, indicated by high LVESP; 2) increased contractility, shown by a leftward shift of end-systolic pressure-volume relationship (ESPVR) with a steepened slope (end-systolic elastance [Ees]), and increased maximum rate of LV pressure rise (dP/dt_max_); and 3) prolonged duration of systole (dTes).^12^ This transient effect is reversible when afterload normalizes.

In this study, we investigate the hemodynamic signature of the Anrep effect—increased contractility and prolonged systole in response to elevated afterload from LVOTO—and its reversibility in HOCM patients, both before and after undergoing PTSMA to reduce the LVOT gradient. By revisiting invasive pressure-volume (PV) data from Steendijk et al,^5^ we analyzed LV function in 14 HOCM patients, focusing on Anrep signature parameters, mechanical work/efficiency indices (stroke work [SW], potential energy [PE], and PV area), and diastolic compliance metrics. A Central Illustration provides a visual overview of the study methodology and key findings.

**Central Illustration:**
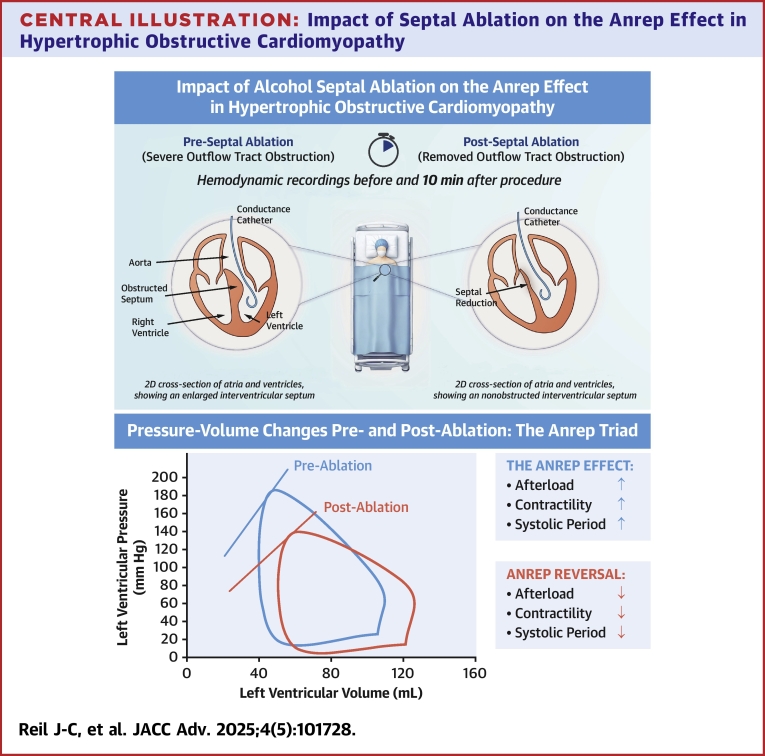
Impact of Septal Ablation on the Anrep Effect in Hypertrophic Obstructive Cardiomyopathy This figure illustrates the hemodynamic changes in HOCM preseptal and postseptal ablation. The left panel shows preablation with obstructed septum and elevated afterload, leading to activation of the Anrep effect (increased contractility and prolonged systolic period). The right panel shows postseptal ablation, where obstruction is reduced, reversing the Anrep effect, improving mechanical efficiency, and lowering cardiac workload. The pressure-volume loop graph compares preablation (in blue) and postablation (in red) hemodynamics, emphasizing reductions in afterload, contractility, and systolic duration. Abbreviation as in Figure 1.

## Methods

### Patient population

This retrospective study, based on data from Steendijk et al,^5^ was conducted at the Thoraxcenter at Erasmus Medical Center in Rotterdam, the Netherlands. The study was approved by the Institutional Review Board of Erasmus Medical Center, and all patients provided written informed consent before participation. Seventeen individuals diagnosed with HOCM were initially enrolled and underwent PTSMA after receiving optimal medical treatment for over 6 months. Diagnosis of HOCM was confirmed through clinical, echocardiographic, and angiocardiographic assessments. Eligibility criteria included patients in NYHA functional class II-IV with significant LVOTO, characterized by a systolic gradient over 50 mm Hg at rest or during provocation, septal thickness above 15 mm, SAM of the mitral valve, and mild or less mitral regurgitation. Two patients exhibited increased LVOT gradients exceeding 50 mm Hg only during exercise or the Valsalva maneuver. The cohort included 2 cases of familial HOCM and 4 with presumed hypertension. Baseline clinical and hemodynamic characteristics are presented in Tables 1 and 2.

**Table 1:** Baseline Characteristics of HOCM Patients (N = 17)

Characteristic	Median [Q1 -Q3] or n (%)
Demographics	
Age, y	60 (52-72)
Male	11 (64%)
Female	6 (36%)
Symptoms	
Angina	6 (35%)
Dyspnea	8 (47%)
Syncope	3 (18%)
Medications	
β-blockers	9 (53%)
Calcium channel antagonists	10 (59%)
Diuretics	6 (35%)
NYHA functional class	
IV	0 (0%)
III	8 (47%)
II	9 (53%)
Echocardiographic parameters	
LVOT gradient (mm Hg)	80 (20-100)
Maximal LV wall thickness (mm)	21.5 (18.5-25)
Ejection fraction (%)	77 (71-86)
SAM severity	
Severe	10 (58.8%)
Moderate	3 (17.6%)
Mild	3 (17.6%)
Nonexisting	1 (6%)

Values are median (IQR) or n (%). SAM severity is categorized as follows: mild (brief SAM without septal contact), moderate (SAM with septal contact lasting less than one-third of the systolic phase), and severe (SAM with septal contact exceeding one-third of systole). Adapted from *Heart*, Steendijk P et al, Volume 94, Issue 10, Pages 1,318 to 1,322, Copyright ©2008, with permission from BMJ Publishing Group Ltd.

HOCM = hypertrophic obstructive cardiomyopathy; LV = left ventricular; LVOT gradient = left ventricular outflow tract gradient; SAM = systolic anterior motion of the mitral valve; NYHA = New York Heart Association.

**Table 2:** Changes in Key Hemodynamic Parameters in HOCM Patients Pre- and Post-PTSMA (N = 14)

	Pre-PTSMA	Post-PTSMA	*P* Value
General			
Heart rate (beats/min)	65 (59-79)	67 (59-79)	0.82
Ejection fraction (%)	69 (62-74)	59 (52-66)	0.0007
Stroke volume (mL)	60 (51-90)	66 (49-81)	0.37
Afterload			
LVOT gradient (mm Hg)	80 (20-100)	0 (0-30)	0.001
ESP (mm Hg)	180 (168-210)	138 (121-160)	0.0001
Ea (mm Hg/mL)	2.5 (1.9-3.0)	1.9 (1.4-2.4)	0.002
Contractility			
Ees (mm Hg/mL)	2.0 (1.7-3.2)	1.5 (0.9-2.2)	0.0001
dP/dt_max_ (mm Hg/s)	1,775 (1,670-1,882)	1,560 (1,309-1,816)	0.017
ESV_150_ (mL)	24 (16-35)	47 (40-72)	0.0006
P_iso_ (mm Hg)	268 (231-304)	217 (191-233)	0.0007
Systolic duration			
dTes (ms)	371 (363-393)	327 (307-367)	0.002
dTes_c_ (ms)	402 (363-412)	346 (325-367)	0.002
dTdPdt (ms)	362 (331-396)	344 (307-372)	0.02
dTdPdt_c_ (ms)	381 (343-404)	340 (321-395)	0.02
Energy and efficiency			
SW (mm Hg·mL)	8,161 (6,892-11,462)	7,495 (5,643-10,374)	0.004
PE (mm Hg·mL)	7,837 (4,990-18,925)	4,915 (3,637-8,621)	0.002
PVA (mm Hg·mL)	16,135 (12,345-33,075)	11,742 (10,101-18,996)	0.0002
ME (SW/PVA) (%)	50 (38-59)	59 (55-63)	0.03
Diastolic function			
EDP (mm Hg)	25 (18-27)	19 (13-26)	0.07
EDV (mL)	96 (76-137)	109 (96-140)	0.01
EDV_15_ (mL)	73 (60-116)	112 (76-135)	0.002
dP/dt_min_ (mm Hg/s)	−1,646 (−1,891 to −1,448)	−1,321 (−1,549 to −1,167)	0.02

Values are median (IQR). Adapted from *Heart*, Steendijk P et al, Volume 94, Issue 10, Pages 1,318 to 1,322, Copyright ©2008, with permission from BMJ Publishing Group Ltd.

dP/dt_max_ = maximum rate of pressure rise; dP/dt_min_ = minimum rate of pressure decrease; dTdPdt = time between dP/dt_max_ and dP/dt_min_; dTdPdt_c_ = dTdPdt corrected for heart rate; dTes = duration of systole; dTes_c_ = duration of systole corrected for heart rate; Ea = effective arterial elastance; Ees = end-systolic elastance; EDP = left ventricular end-diastolic pressure; EDV = end-diastolic volume; EDV_15_ = left ventricular end-diastolic volume at a pressure of 15 mm Hg; ESP = left ventricular end-systolic pressure; ESV_150_ = end-systolic volume at 150 mm Hg; ME = mechanical efficiency (SW/PVA ratio); PE = potential energy; P_iso_ = isometric pressure maximum; PTSMA = percutaneous transluminal septal myocardial ablation; PVA = pressure-volume area; SW = stroke work; other abbreviations as in Table 1.

### Septal ablation procedure

The PTSMA procedure was performed uniformly across all participants as described in Steendijk et al.^5^ Initially, a 6F transfemoral pacemaker was placed in the right ventricle for all patients. Baseline hemodynamic data, including LVOT gradient assessments, were recorded at rest. The procedure involved using a 6F percutaneous coronary angioplasty guiding catheter for selective vessel probing, guided by intraprocedural myocardial contrast transthoracic echocardiography. After confirming appropriate vessel closure by balloon inflation, 1 to 2 mL of alcohol was injected slowly through the balloon catheter lumen under continuous transthoracic echocardiography imaging to reduce the LVOT gradient. Care was taken to prevent alcohol leakage into major coronary branches. Hemodynamic measurements were repeated within 10 minutes postprocedure, and pacemaker leads were monitored for at least 48 hours to ensure patient safety.

### Hemodynamics and LV function

**Figure 1:**
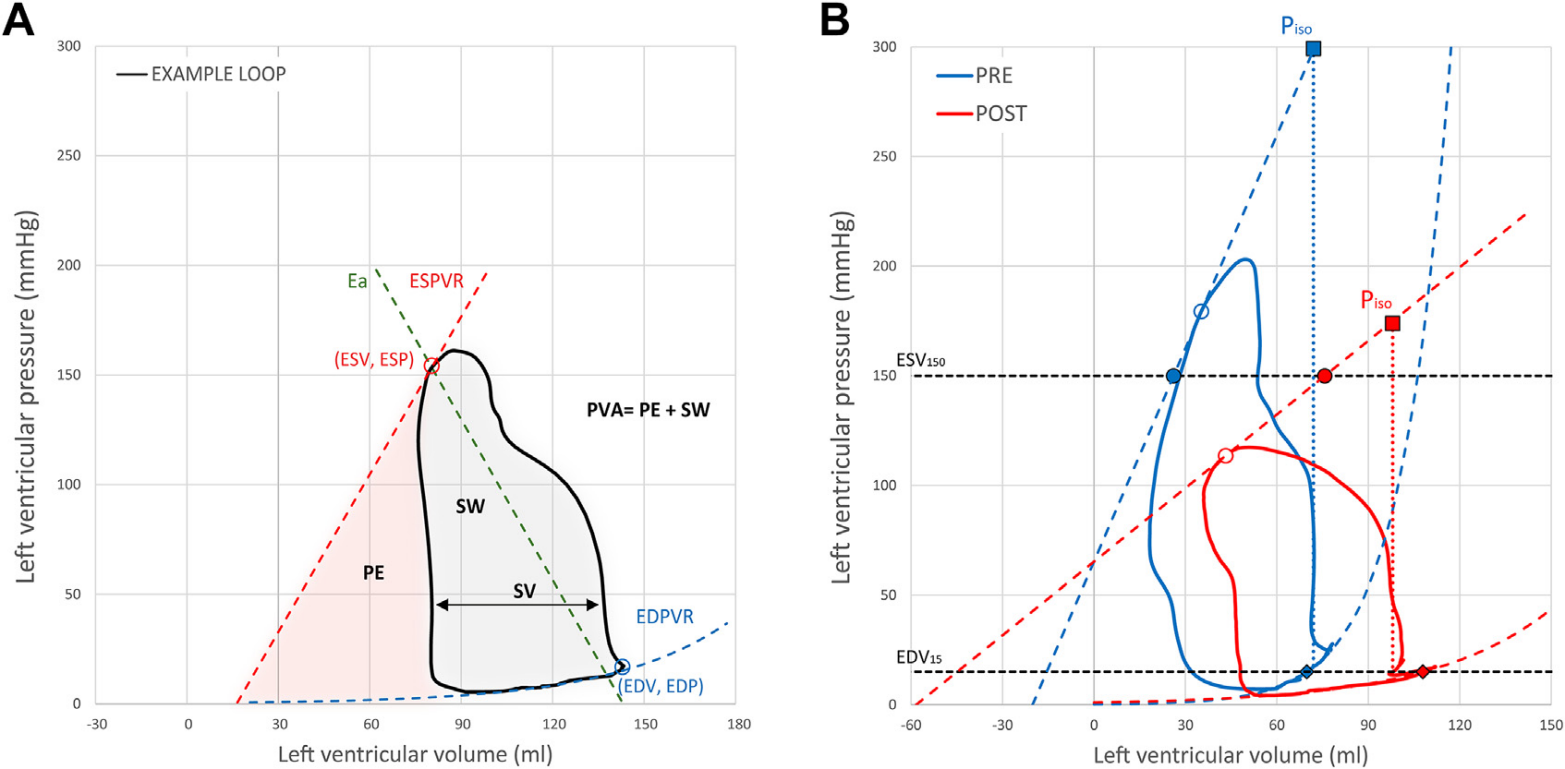
Hemodynamic Responses in Hypertrophic Obstructive Cardiomyopathy Preseptal/Postseptal Ablation (A) Typical pressure-volume loop (black) outlines key indices of LV function such as ESPVR (dashed red-line), EDPVR (dashed blue-line), and Ea (dashed green-line), along with SV, SW, PE, and total PV area (PVA = PE + SW). (B) Representative pressure-volume loops from a HOCM patient preseptal (blue) and postseptal ablation (red), with a postprocedure rightward shift in ESPVR and increased ESV_15_ (black dashed line and solid red circle), indicating reduced contractility, and a rightward EDPVR shift with higher EDV_15_ (black dashed line and red diamond), denoting augmented diastolic capacity. Theoretical isovolumic pressure-volume loops (blue- and red-dotted lines) highlight the Anrep's role in offsetting increased afterload. Symbols represent measurement points: open circles for ESV and ESP, solid circles for ESV_150_, diamonds for EDV_15_, and squares for P_iso_. Black dashed lines denote pressures of 150 and 15 mm Hg, while vertical dashes visualize theoretical isovolumic contractions. EDPVR, End-Diastolic Pressure-volume Relationship; EDV_15_ = left ventricular end-diastolic volume at a pressure of 15 mm Hg; ESP = left ventricular end-systolic pressure; ESPVR = end-systolic pressure-volume relationship; ESV_150_ = end-systolic volume 150 mm Hg; HOCM = hypertrophic obstructive cardiomyopathy; LV = left ventricular; PE = potential energy; P_iso_ = isometric pressure maximum; PV = pressure-volume; PVA = pressure-volume area; SW = stroke work.

**Figure 2:**
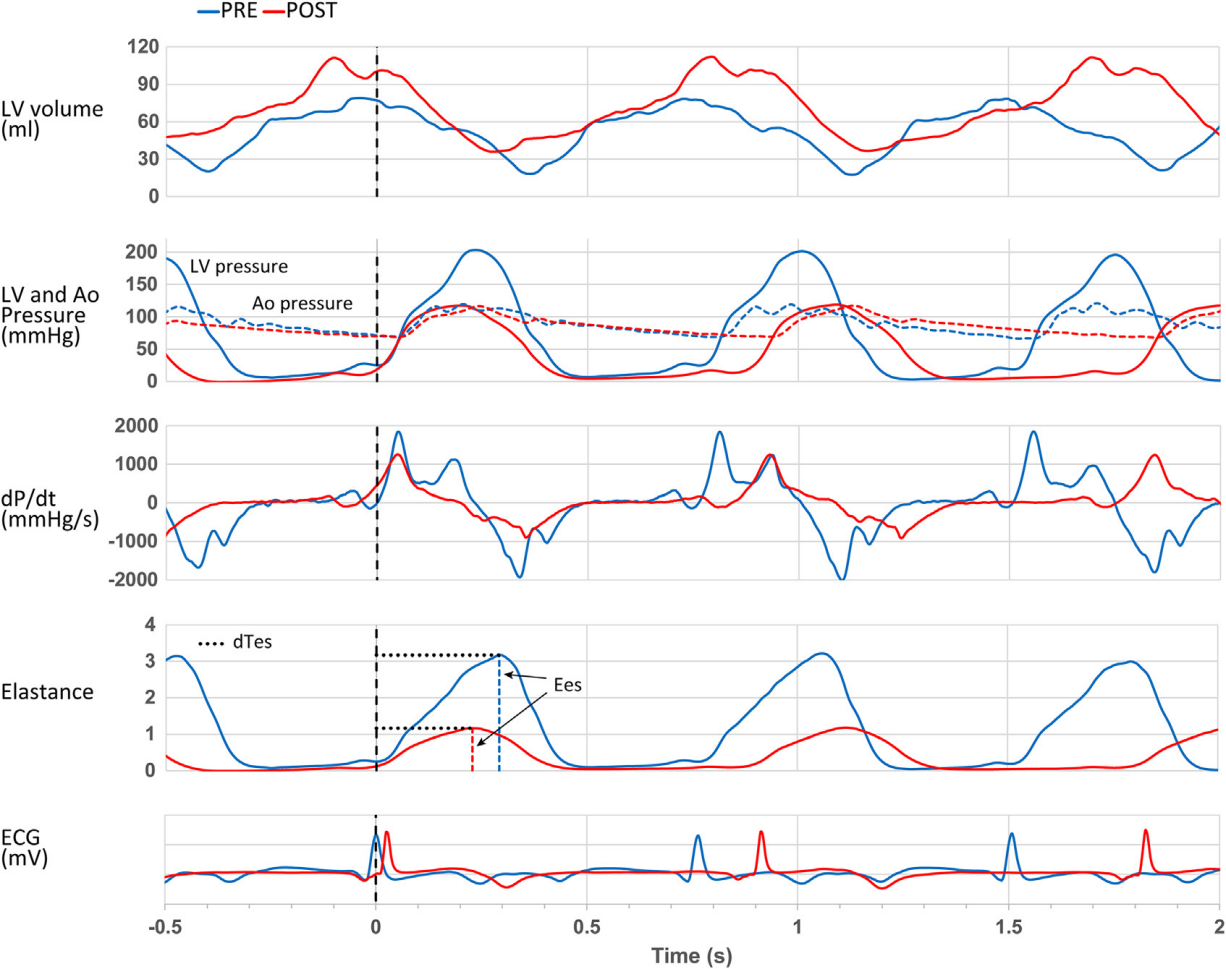
Hemodynamic Analysis in Hypertrophic Obstructive Cardiomyopathy Preseptal/Postseptal Ablation Shown are preseptal (blue) and postseptal ablation (red) traces of volume-time, left ventricle (LV) and aortic (Ao) pressure-time, dP/dt, elastance-time, and electrocardiograms (ECG). Synchronization of preseptal and postseptal ablation traces at the onset of the QRS complex in the ECG is marked by the dashed black line at t = 0 (the second beat of both examples). The figure demonstrates, postseptal ablation, reductions in dP/dt_min_ and dP/dt_max_, a decrease in end-systolic elastance (Ees, depicted with dashed vertical red lines), and a shortened time to reach end-systolic elastance (dTes, depicted with horizontal dotted black lines). The aortic and LV pressure traces illustrate the removal of the LV-aortic pressure gradient through septal ablation, highlighting the procedural success in alleviating hemodynamic obstruction. Adapted from *Heart*, Steendijk P et al, Volume 94, Issue 10, Pages 1,318 to 1,322, Copyright ©2008, with permission from BMJ Publishing Group Ltd. dP/dt_max_ = maximum rate of pressure rise; dP/dt_min_ = minimum rate of pressure decrease.

LV function in the initially enrolled 17 HOCM patients undergoing PTSMA was assessed using PV loops obtained via 7F combined pressure-conductance catheters (CD Leycom) connected to a Cardiac Function Lab (CFL-512, CD Leycom). Steady-state loops, recorded pre- and acutely post-PTSMA (<10 min), were calibrated to echocardiographically determined end-diastolic (EDV) and end-systolic (ESV) volumes, as well as stroke volume.^5^ For an illustrative representation of a typical PV loop, illustrating key concepts of LV mechanics, refer to Figure 1A. The ESPVR was determined using a single-beat estimation, characterized by its slope (Ees) and volume axis intersection (V_0_), with contractility inferred from these attributes.^5^ The LV end-systolic volume at a predefined LVESP of 150 mm Hg (ESV_150_, denoted by the black horizontal dashed line) was calculated by integrating the ESPVR slope and volume intercept (Figure 1B). Diastolic function was assessed by calculating LV diastolic capacity (LVEDV_15_, marked by the black horizontal dashed line) as the EDV at a predefined LV end-diastolic pressure of 15 mm Hg (Figure 1B). This was calculated using a single-beat end-diastolic PV relationship (EDPVR) with an exponential fit using the equation LV end-diastolic pressure = k×e^(β×LVEDV)^, where β is the chamber stiffness coefficient and k is the fitting constant. LV diastolic capacity was then calculated as: LVEDV_15_ = Ln (15)/(k×β), according to Ten Brinke et al.^13^ The time-varying elastance approach was adopted to examine the temporal evolution of LV contractility. Instantaneous elastance, E(t), was calculated for each PV loop point by connecting V_0_ to that specific PV point: E(t) = P(t)/[V(t)-V_0_]. Elastance-time curves were plotted from each heartbeat, with peak values denoted as Ees, defining end-systole (Figure 2). The dTes, the time interval from end-diastole (marked by the R-wave of the QRS complex) to end-systole, represents the entire systolic phase, including both isovolumic contraction and ejection phases (Figure 2). This interval is less load-dependent than previously used indices such as systolic ejection time.^14^ Additionally, the time between dP/dt_max_ and dP/dt_min_ (dTdPdt) was assessed as another method to evaluate the systolic period (Figure 2). In patients with HOCM, the pressure rise and decay often exhibit a biphasic pattern. When the dP/dt curve showed biphasic behavior, the interval between the first positive peak of dP/dt_max_ and the second negative peak of dP/dt_min_ was measured. To account for heart rate influence on systolic duration, the Fridericia formula for QT interval correction (QT_c_ = QT/[RR^1/3^]) was applied to all data, standardizing systolic duration to a heart rate of 60 bpm.^14^ This provided heart rate-corrected durations for both dTes and dTdPdt, termed dTes_c_ and dTdPdt_c_, respectively. Indicators of contractility included Ees, ESV_150_, and dP/dt_max_. LVESP and effective arterial elastance (Ea) were used as indicators of the afterload. LV SW is the area surrounded by the LV PV loop, while PE is the triangle bordered by the ESPVR, the volume axis, and the LV PV loop (Figure 1A). Total PV area (PVA = PE + SW) correlates well with myocardial oxygen consumption,^15^ and LV mechanical efficiency was defined by SW/PVA.

### Statistics

Given the small sample size and the assumption of non-normal distribution, all continuous variables were analyzed using the nonparametric Wilcoxon signed rank test. Continuous variables are reported as median with first (Q1) and third (Q3) quartiles. A *P* value <0.05 was considered statistical significance. Analyses were performed using GraphPad Prism 8.1.2.

## Results

### Patient clinical characteristics

In this study, 17 patients with HOCM underwent PTSMA to relieve LVOTO. Baseline clinical characteristics are presented in Table 1. The cohort had a median age of 60 (52-72) years, with 6 patients reporting angina, 3 syncope, and 8 dyspnea. Despite optimal medical therapy (β-blockers, calcium channel blockers, diuretics), 8 patients were NYHA functional class III and 9 patients were class II (Table 1). Preprocedural echocardiographic assessments confirmed significant LVOTO in all patients: resting gradient 80 (20-100) mm Hg, septal thickness 21.5 (18.5-25) mm, SAM classified as severe or moderate in 13 of 17 cases, and mild mitral regurgitation in all cases (Table 1). Postprocedure, LVOT gradient decreased to 0 (0-30) mm Hg (from 80 [IQR: 20-100], *P* = 0.001), SAM resolved or was reduced to mild, and mitral regurgitation was no longer detectable.

### Hemodynamic changes in HOCM: activation and reversal of the Anrep effect

LV function was assessed by invasive PV catheterization in all 17 HOCM patients. However, 3 individuals were excluded from the final analysis: 2 due to insufficient afterload reduction post-PTSMA at rest (a key criterion for assessing the procedure's efficacy) and elevated LVOT gradients appearing only during exercise or Valsalva maneuvers, and one due to poor PV signal quality. Preprocedure and postprocedure hemodynamic parameters of the 14 included patients are summarized in Table 2. Representative PV loops from a patient pre- (blue) and post-PTSMA (red) are shown in Figure 1B.

**Figure 3:**
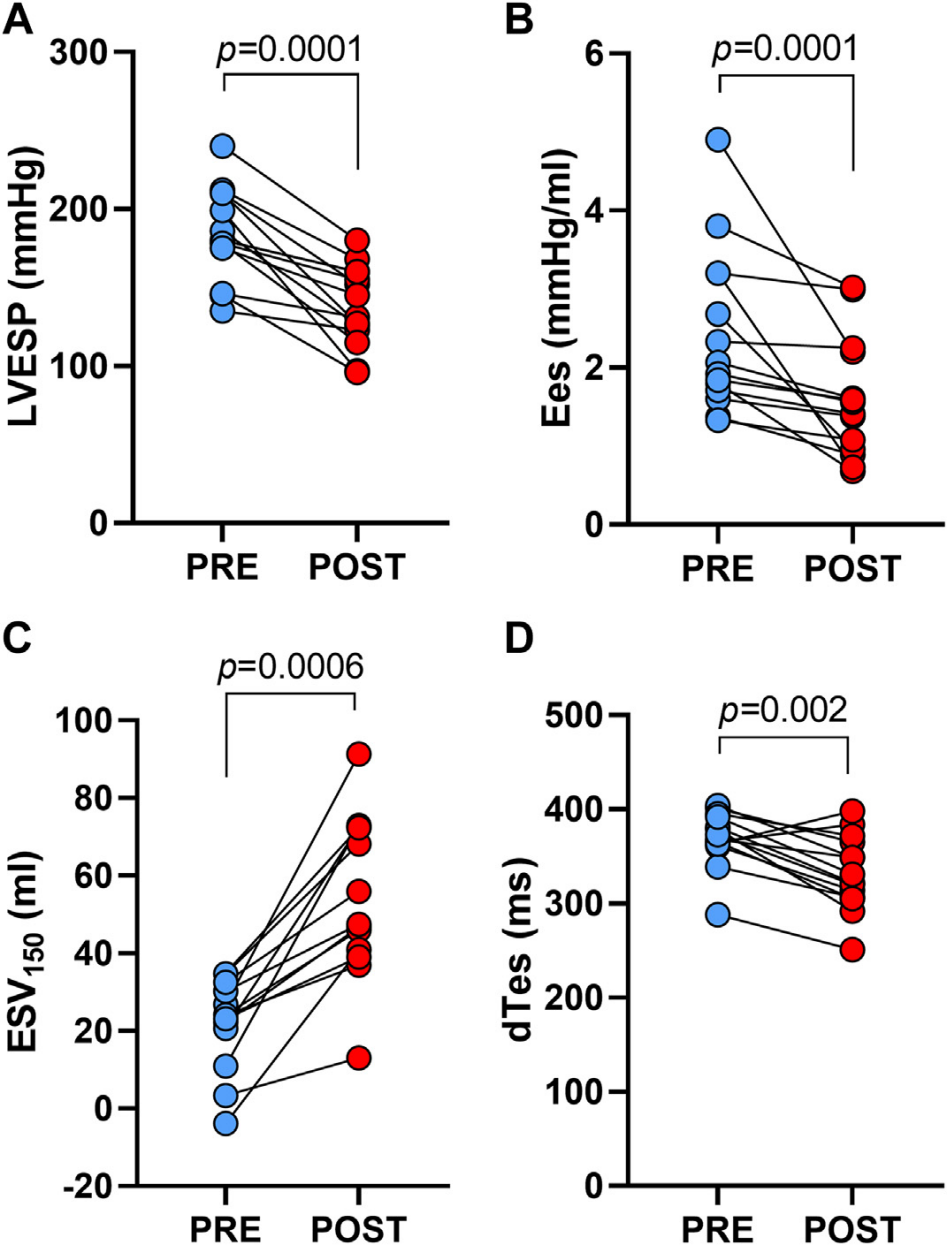
Hemodynamic Changes in Hypertrophic Obstructive Cardiomyopathy Preseptal/Postseptal Ablation (A) Left ventricular end-systolic pressure (LVESP) shows the pressure within the LV at the end of systole. (B) End-systolic elastance (Ees) represents the stiffness of the LV at the end of systole. (C) End-systolic volume 150 mm Hg (ESV_150_) quantifies the volume of the LV when the pressure is at 150 mm Hg, indicating afterload resistance. (D) The duration of systole (dTes) measures the total time span of the systolic phase of the cardiac cycle. Asterisks (∗) indicate statistical significance, highlighting the hemodynamic improvement postseptal ablation. Adapted from *Heart*, Steendijk P et al, Volume 94, Issue 10, Pages 1,318 to 1,322, Copyright ©2008, with permission from BMJ Publishing Group Ltd.

The Anrep Triad—comprising elevated afterload (LVESP, Ea), increased contractility (Ees, dP/dt_max_), and prolonged systole (dTes, dTdP/dt)—was assessed through invasive comparison of pre- and post-PTSMA hemodynamics. Postprocedure, afterload reduction paralleled the decline in resting LVOT gradient: LVESP decreased from 180 (IQR: 168-210) to 138 (IQR: 121-160) mm Hg (*P* = 0.0001), and Ea from 2.5 (IQR: 1.9-3.0) to 1.9 (IQR: 1.4-2.4) mm Hg/mL (*P* = 0.002) (Figures 1B, 2, and 3A, Table 2). Concomitantly, contractility decreased, evidenced by a rightward shift in the ESPVR (Figure 1B), a drop in Ees from 2.0 (IQR: 1.7-3.2) to 1.5 (0.9-2.2) mm Hg/mL (*P* = 0.0001) (Figures 2 and 3B), and an increase in ESV_150_ from 24 (IQR: 16-35) to 47 (IQR: 40-72) mL (*P* = 0.0006) (Figure 1B, solid red circle; Figure 3C and Table 2). Additionally, dP/dt_max_ decreased from 1,775 (IQR: 1,670-1,882) to 1,560 (IQR: 1,309-1,816) (*P* = 0.017) (Table 2, Figure 2). Systolic duration also shortened post-PTSMA: dTes decreased from 371 (IQR: 363-393) to 327 (IQR: 307-367) ms (*P* = 0.002), and dTdPdt from 362 (IQR: 331-396) to 344 (IQR: 307-372) ms (*P* = 0.02) (Table 2, Figure 3D). Heart rate-corrected durations for both dTes and dTdPdt validated the shortening of the systolic period (dTes_c_: 402 [IQR: 363-412] to 346 [IQR: 325-367] ms, *P* = 0.002; dTdPdt_c_: 381 [IQR: 343-404] to 340 [IQR: 321-395] ms, *P* = 0.02) (Table 2).

Collectively, these changes confirm that the Anrep mechanism, typically transient in healthy hearts, becomes chronically activated in HOCM (eg, sustained over months to years) due to continuous high afterload from LVOTO. This sustained activation is shown by persistently elevated baseline parameters (eg, LVESP, Ea, Ees, dP/dt_max_, dTes) prior to PTSMA. Notably, LVOTO relief reversed this chronic activation of the Anrep effect.

### Energetic and functional consequences of Anrep reversal

Anrep activation in HOCM, while a compensatory response to maintain stroke volume under high afterload, comes at a high energetic cost. Preprocedure, HOCM patients exhibited an elevated mechanical workload, which significantly decreased following Anrep effect reversal postprocedure. Post-PTSMA, myocardial SW declined from 8,161 (IQR: 6,892-11,462) to 7,495 (IQR: 5,643-10,374) mm Hg·mL (*P* = 0.004), PE from 7,837 (4,990-18,925) to 4,915 (IQR: 3,637-8,621) mm Hg·mL (*P* = 0.002), and total PVA from 16,135 (IQR: 12,345-33,075) to 11,742 (IQR: 10,101-18,996) mm Hg·mL (*P* = 0.0002) (Table 2). This reduction in workload was paralleled by an improvement in mechanical efficiency, reflected by a rise in SW/PVA from 50 (IQR: 38-59) to 59% (IQR: 55%-63%) (*P* = 0.03) (Table 2). Although ejection fraction decreased postprocedure (69% [IQR: 62%-74%] to 59% [IQR: 52%-66%], *P* = 0.0007), stroke volume remained unaffected (60 [IQR: 51-90] mL vs 66 [IQR: 49-81] mL, *P* = 0.37) (Table 2). Additionally, -dP/dt_min_ decreased post-PTSMA from −1,646 (IQR: −1,891 to −1,448) mm Hg/s to −1,321 (IQR: −1,549 to −1,167) mm Hg/s (*P* = 0.02) (Table 2, Figure 2), suggesting a slight prolongation in myocardial relaxation. However, this was accompanied by a rightward shift of the EDPVR and an increase in EDV_15_ from 73 (IQR: 60-116) mL to 112 (IQR: 76-135) mL (*P* = 0.002) (Figure 1B, solid red diamond; Table 2), and a higher EDV at a trending lower EDP (25 [IQR: 18-27] mm Hg to 19 [IQR: 13-26] mm Hg; *P* = 0.07), indicating improved diastolic compliance. These findings support enhanced ventricular filling and attenuation of diastolic dysfunction following septal reduction therapy.

## Discussion

**Figure 4:**
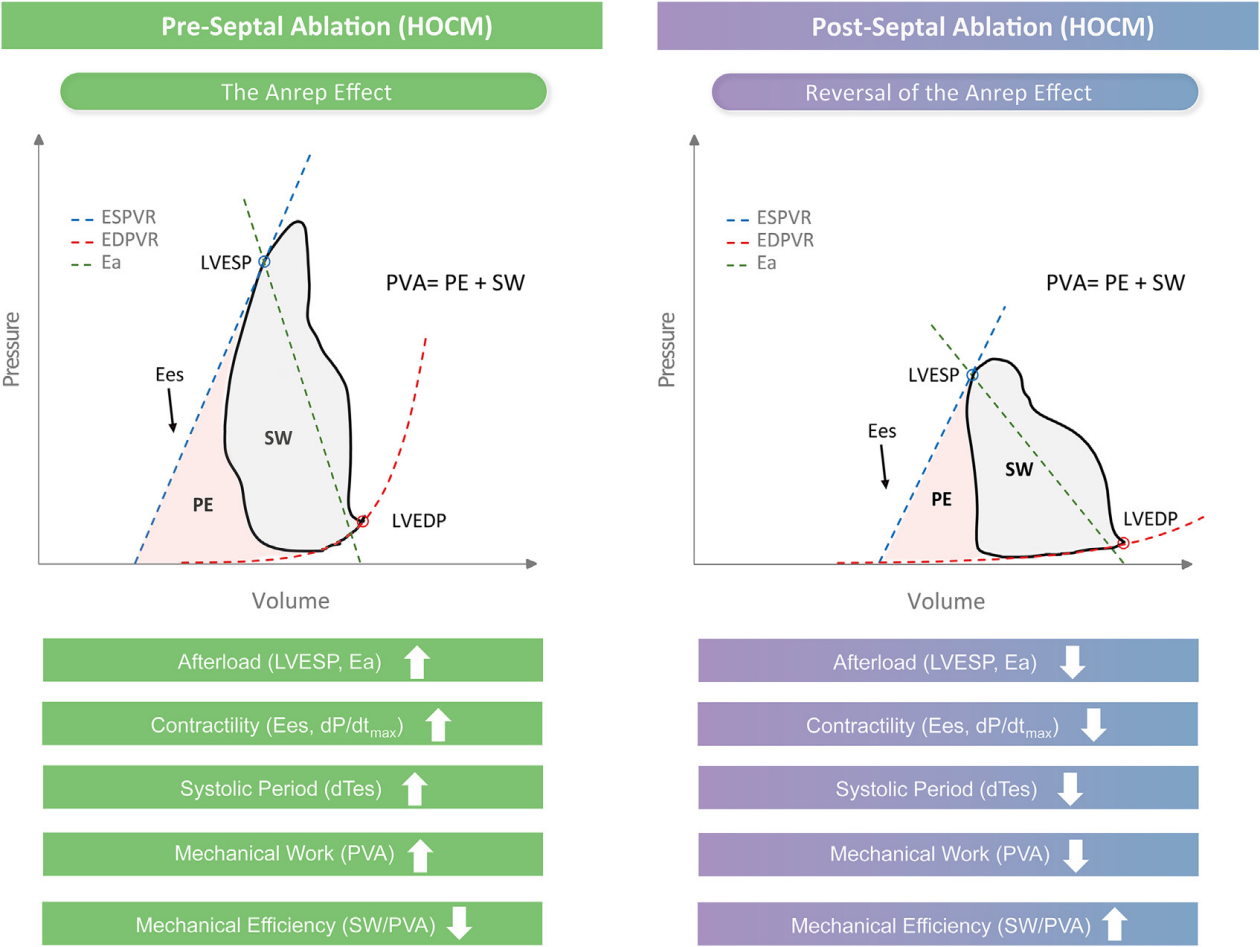
The Anrep Effect and Its Reversal in Hypertrophic Obstructive Cardiomyopathy Postseptal Ablation This conceptual diagram synthesizes the study's main findings, showcasing the Anrep effect’s role in maintaining cardiac function under increased afterload (left) and its reversal postseptal ablation (right) in HOCM. Key hemodynamic measurements included left ventricular (LV) end-systolic pressure (LVESP) and effective arterial elastance (Ea) as afterload indicators, and contractility indices such as end-systolic elastance (Ees), the maximum rate of LV pressure rise (dP/dt_max_), and the duration of systole (dTes). Stroke work (SW), potential energy (PE), and total pressure-volume area (PVA) quantified mechanical work and efficiency (SW/PVA). Postablation, LVESP, Ea, Ees, dP/dt_max_, dTes, SW, PE, and PVA were all significantly reduced, indicating decreases in afterload, contractility, and mechanical work, leading to improved cardiac efficiency and function. Symbols: ↑ Increase, ↓ Decrease. Abbreviations as in Figure 1.

This study advances prior evidence of chronic Anrep effect activation in HOCM, previously detected via noninvasive echocardiography.^9^ We applied invasive pressure-volumetry analysis, recognized as the definitive standard for evaluating cardiac function through direct measurements of ventricular pressures and volumes. This approach overcomes the geometric assumptions inherent in echocardiographic methods, providing greater precision in assessing cardiac mechanics and energy dynamics. Using a new cohort of 14 HOCM patients, we confirmed the sustained activation of the Anrep effect and documented its rapid reversal following PTSMA. This finding supports the effect’s sensitivity to afterload reduction and its role as a dynamic compensatory mechanism. Additionally, we introduce the concept of the *Anrep Triad*, a novel hemodynamic signature comprising 3 core features: elevated afterload (increased LVESP and Ea), enhanced contractility (leftward shift of ESPVR, steeper Ees slope, higher dP/dt_max_), and a prolonged systolic phase (dTes, dTdPdt) (Figure 2, Table 2). Key findings are summarized in Figure 4.

Activation of the Anrep effect in HOCM imposes a heavy mechanical workload (higher SW, PE, and PVA) and leads to a reduction in LV capacity, signaling diminished mechanical efficiency and high energy consumption. Moreover, the Anrep effect contributes to a deterioration in LV diastolic filling, as evidenced by the leftward shift of the EDPVR and a lower EDV_15_ (see the blue PV loop in Figure 1B and Table 2). These findings support the notion that, while the Anrep effect is an adaptive response to increased afterload, it is energetically costly and imposes significant mechanical stress on the myocardium. Under physiological conditions, the transient, energy-intensive nature of the Anrep effect is balanced by efficient energy delivery, optimal coronary flow reserve, ample oxygen supply, and well-functioning energy recycling systems. However, in HOCM, this balance becomes progressively disturbed by changes in mitochondrial energy substrates^16^^,^^17^ and mitochondrial dysfunction,^18^ limited myocardial perfusion,^19^ and reduced coronary flow reserve.^20^ These changes are compounded by a decrease in phosphocreatine/ATP ratios at rest,^21^ which worsens under physical stress,^22^ and decreased cardiac work efficiency, further exacerbated by LVOTO.^6^ These observations align with the chronic activation of the Anrep effect we document, underscoring that while the Anrep effect augments LV contractility in response to high afterload, its prolonged, energy-intensive nature *exhausts* energy reserves and reduces mechanical work efficiency if not supported by adequate systemic metabolic health.

### Reversing the Anrep effect: impact of PTSMA

PTSMA offers an effective intervention to reduce LVOTO and alleviate the mechanical and energetic demands imposed by the Anrep effect. This is evidenced by significant reductions in afterload (LVESP, Ea) and key contractility parameters (dTes and dTdPdt, Ees, and dP/dt_max_), alongside decreases in cardiac workload (SW, PE, and PVA), ultimately leading to improved mechanical efficiency (Table 2). Importantly, the reversal of the Anrep Triad occurs independent of confounding variations in catecholamines, heart rate, the Gregg effect, preload adjustments, or myocardial damage. A decrease in catecholamine levels, for instance, would be expected to reduce contractility and prolong systole,^23^ a trend that was not observed in our data. Similarly, the unchanged heart rate before and after PTSMA rules out any influence of the Bowditch effect (Table 2). While microvascular dysfunction^19^ and reduced coronary flow reserve^20^ are known to improve partially with LVOTO relief,^24^ their impact on contractility in this context (the Gregg effect) is likely minimal, as coronary perfusion remains within the autoregulatory range and is not expected to significantly influence contractility in blood-perfused tissues.^25^ But any potential improvement in contractility from enhanced coronary perfusion would likely work to offset, rather than fully counteract, the decrease in contractility observed postprocedure. Additionally, the potential role of the slow force response, marked by delayed contractility increases after an elevation in EDV post-PTSMA, would only serve to lessen the reduction in contractility following Anrep effect reversal.^12^ An increase in EDV post-PTSMA could likewise recruit the Frank-Starling mechanism, preserving stroke volume and offsetting any decrease in contractility (Figure 1B). Given that our hemodynamic measurements were taken within 10 minutes post-PTSMA, before inflammatory responses could peak (typically 3 hours postinjury),^26^^,^^27^ it is unlikely that negative inotropic influence resulting from inflammatory responses impacted our findings. Even in the presence of localized myocardial injury, the targeted nature of PTSMA, which induces scar formation specifically in the septal region, helps limit the extent of functional impairment.^28^ Thus, PTSMA is unlikely to impair global LV function.^28^ Moreover, continued administration of β-blockers, calcium channel antagonists, and diuretics post-PTSMA ensures uniform accounting for any confounding pharmacological effects, allowing for a clearer understanding of the procedure's impact on Anrep-related hemodynamics. Collectively, these factors strongly suggest that the observed improvements post-PTSMA are directly attributable to afterload reduction and the reversal of the Anrep effect, independent of external confounding influences such as catecholamines, preload variations, tissue inflammation, or pharmacological interventions.

### Molecular mechanisms responsible for the Anrep effect in HOCM

Understanding the molecular basis of the Anrep Triad is key to explaining its high mechanical and energetic requirements and the efficacy of interventions like PTSMA in improving mechanical efficiency in HOCM. The Anrep effect allows the heart to sustain contractility under elevated afterload by recruiting myosin motors from a normally inactive reserve within cardiomyocytes.^9^ Approximately 55% of sarcomeric myosin heads are held in this energy-conserving inactive state (reserve pool), minimizing ATP consumption.^9^ When afterload increases, a greater proportion of these myosin heads transition from inactivity to activation, making them available for actin binding and force generation; this transition correlates with the activation of the Anrep effect. This process may involve a calcium feedback mechanism that responds to afterload, dynamically adjusting calcium availability to sustain sarcomere activation and force production under elevated wall stress.^29^

In HOCM, the Anrep effect, normally transient and adaptive, undergoes chronic overactivation. The persistent elevation in afterload caused by LVOTO disrupts the equilibrium between inactive and active myosin states, leading to excessive myosin recruitment and sustained hypercontractility.^9^ The activation of the Anrep effect in HOCM is characterized by:

1. Afterload-sensitive (myosin recruitment) phase: Prolonged wall stress from sustained afterload elevation due to LVOTO continuously activates dormant myosin heads from the reserve pool, while prolonging the duration of myosin-actin (cross-bridge) interactions.^9^ This process amplifies LV contractility, extends the duration of systole, and raises energy demand, hallmarks of Anrep effect activation observed in obstructive HCM (Table 2).
2. Pathogenic-dependent (myosin recruitment) phase: An estimated 20% of myosin heads are recruited from the inactive reserve pool due to sarcomeric pathogenic variants, post-translational modifications (eg, phosphorylation of myosin-binding protein C or myosin light chain), or shifts in myocardial energetics, particularly a decrease in the ATP/ADP ratio.^30^^,^^31^ These molecular events *compound* the activation of the Anrep effect by destabilizing myosin’s resting configuration, causing excessive myosin-actin engagement.^9^ This establishes a “baseline” level of hypercontractility, already observed in nonobstructive HCM.^30^^,^^31^

The combined activation of these processes explains why the Anrep effect is both energetically costly and reversible in HOCM. An increased number of active myosin motors raises the energetic cost of contraction,^32^ depletes myocardial energy reserves,^21^^,^^22^ and elevates SW, PE, and PVA (Table 2). Post-PTSMA, these (energy-consuming) metrics decrease as afterload is reduced, allowing myosin heads to return to their dormant (energy-efficient) state, reducing contractility, shortening systole, and improving mechanical efficiency (Table 2). The observed improvements in diastolic compliance, indicated by an increased EDV at a trending lower EDP (*P* = 0.07) (Table 2), support this deactivation of myosin, reducing myocardial stiffness and enhancing ventricular filling. This allows the heart to recruit the Frank-Starling reserve, maintaining stroke volume *despite* the limited capacity of preload to augment contraction in HCM (Figure 1B, Table 2).^31^^,^^33^ Future research should investigate the molecular dynamics of the Anrep Triad, particularly focusing on noninvasive therapies targeting this energy-consuming process. Preclinical and clinical studies on myosin inhibitors, such as mavacamten, support this concept by demonstrating their ability to lower hypercontractility, reduce cellular energy consumption,^34^^,^^35^ and improve LVOT gradients and diastolic function.^36^, ^37^, ^38^

### Anrep mismatch: reserve depletion and the progression to end-stage HCM

In HCM, ejection fraction is typically normal or hyperdynamic (>70%), even before the onset of hypertrophy.^39^^,^^40^ However, 5% to 15% of patients progress to LV hypokinesia, where ejection fraction falls below 40%. These patients exhibit ventricular dilation, regression of hypertrophy, ischemia, microvascular dysfunction, and diminished coronary reserve.^41^ This advanced stage, known as dilated-hypokinetic or end-stage HCM, is often accompanied by severe pulmonary hypertension in up to 80% of cases.^42^ When aggravated by LVOTO, afterload increases further, exacerbating heart failure risk.^42^ The severity of symptoms and systolic dysfunction in this stage correlates with a reduction in phosphocreatine/ATP ratios, indicating a causal relationship between high mechanical work, ventricular dilation, and energy depletion.^43^ In HCM, high mechanical work and energy insufficiency, rather than microvascular impairment, ischemia, hypertrophy, or fibrosis, are antecedents to the progression of disease.^7^^,^^21^^,^^39^^,^^40^ We suspect that, if left untreated, prolonged exposure to high afterload (LVOTO, hypertension), coupled with excessive drainage of myocardial energy reserves,^22^ leads to exhaustion of the Anrep reserve, resulting in LV hypokinesia and the transition to end-stage HCM. This condition, where the heart can no longer maintain stroke volume due to inadequate contractility, aligns with John Ross' concept of “afterload mismatch,” described in the late 1970s.^44^ We extend this idea, introducing the term *Anrep mismatch*, which denotes the depletion of the Anrep reserve or its failure to compensate for long-term high mechanical demands. This mismatch signifies the peak of chronic high afterload, energy exhaustion, and declining LV contractility.

At the cellular level, severe energy depletion under chronic afterload likely induces a state akin to isometric, *rigor-like* contraction—a hypercontracture state.^9^ In this state, myofibrils contract with minimal shortening and reduced mechanical work, characterized by increased and prolonged contact of myosin heads with actin, which bind strongly and for extended periods in a nonrelaxing (noncycling), rigor-like manner.^9^ This prevents extensive shortening, reducing ventricular mechanical work and ejection, while the end-stage HCM heart pathologically dilates, attempting to utilize an already compromised preload (Frank-Starling) reserve.^31^^,^^33^ Our data (Figure 1B) conceptualize this sequence by illustrating the consequences of reintroducing pre-PTSMA afterload levels (180 mm Hg) post-PTSMA. The theoretical isovolumic PV loops, represented by blue and red dotted lines, demonstrate the potential state of the heart before and after the procedure (Figure 1B). If the HCM heart encounters pre-PTSMA levels of afterload post-PTSMA (Figure 1B, blue open circle), purely isovolumetric contractions could occur (red P_iso_, isometric pressure maximum, ∼170 mm Hg). Without sufficient Anrep compensation, the heart would fail to eject any blood, producing no work as the isometric maximum post-PTSMA falls below the pre-PTSMA afterload, preventing aortic valve opening.

Addressing Anrep mismatch requires afterload reduction interventions, such as relieving outflow obstruction in aortic stenosis^45^ or HOCM with acute LV hypokinesia.^46^ These strategies lead to rapid functional recovery by reducing afterload, improving contractility, and restoring ventricular performance. In summary, persistent high afterload and energy depletion diminish myofibrillar efficiency, shifting the heart toward a low-functioning, hypokinetic state, which is a hallmark of Anrep *mismatch*. This state often coexists with diminished adrenergic activation, limited preload reserve,^31^^,^^33^ microvascular impairment, ischemia, or fibrosis, further accelerating LV dysfunction in patients transitioning to *or* already in the end stage of HCM.

### Study limitations

The invasive nature of PV catheterization inherently limited our cohort size (N = 14), reflecting its technical complexity and restricted clinical use in HOCM management. Ethical constraints also precluded the inclusion of a dedicated control group, as invasive catheterization of healthy individuals was not justifiable in this study. Despite these constraints, reference values from healthy individuals with a median age of 65 (62-68) years, as reported by Wachter et al,^47^ provide a useful comparative context. These values, obtained using a similar PV conductance catheter technique, include key Anrep Triad parameters: LVESP (118 [IQR: 111-130] mm Hg), Ea (1.2 [IQR: 0.8-1.7] mm Hg/mL), Ees (1.4 [IQR: 1.26-1.65] mm Hg/mL), dP/dt_max_ (IQR: 1,562 [1,549-1,866] mm Hg/s), and dTes (354 [IQR: 332-374] ms).^47^ A qualitative comparison suggests that our pre-PTSMA HOCM cohort exhibited markedly elevated afterload, increased contractility, and prolonged systole (Table 2). Post-PTSMA, most of our study parameters moved closer to the normative values reported in Wachter et al,^47^ indicating hemodynamic normalization after afterload reduction. Notably, our PV analysis aligns with and extends prior noninvasive observations of the Anrep effect in HOCM,^9^ strengthening the evidence for the Anrep Triad’s role in this pathology. Finally, the absence of genetic profiling, particularly for sarcomeric pathogenic variants common in HCM, limits exploration of genotype-phenotype correlations influencing Anrep activation. Future studies with larger cohorts, incorporating genetic data and noninvasive PV characterization, are needed to validate these relationships and generalize the Anrep Triad across HOCM subpopulations.

## Conclusions

This study comprehensively examines the chronic activation of the Anrep effect in HOCM, underscoring its significant impact on cardiac function and energy dynamics. Through invasive PV analysis of 14 patients undergoing PTSMA, we identified the distinct Anrep Triad, a hemodynamic signature marked by increased contractility and prolonged systole in response to elevated afterload, which is accompanied by heightened mechanical workload and energy consumption. These findings unify numerous clinical and basic research observations, highlighting that key traits of HOCM, such as hyperdynamic systole, exacerbated diastolic dysfunction, and elevated energy demands, are directly linked to Anrep effect activation. The reversal of this effect following LVOTO removal via septal reduction therapy explains the procedure’s success in improving patient outcomes, including symptom relief, enhanced cardiac efficiency, better diastolic function, and improved quality of life. Importantly, the reversal of the Anrep effect post-PTSMA occurs independently of confounding factors like catecholamines, preload variations, inflammation, or pharmacological effects. These results open new avenues for studying the Anrep effect in other cardiac conditions characterized by increased afterload, offering a novel foundation for clinical intervention and future research.

Perspectives **COMPETENCY IN MEDICAL KNOWLEDGE 1:** The chronic activation of the Anrep effect in HOCM redefines our understanding of disease progression, shifting the focus from pathogenic variants alone to the role of pathophysiological adaptation driven by persistent afterload due to LVOTO. This study demonstrates that the Anrep effect, typically a transient contractile response to elevated afterload, becomes a prolonged, energy-consuming mechanism in HOCM. Its reversal through septal reduction therapy highlights the critical need to address afterload in order to restore mechanical efficiency. By reducing contractility and mechanical workload while preserving stroke volume, septal ablation relieves the heart's energy-intensive compensatory burden, leading to improved patient outcomes. This direct link between the Anrep effect and cardiac energy dynamics places myocardial energy depletion as a key factor in HOCM pathophysiology, suggesting that therapies targeting this mechanism could attenuate symptoms and slow disease progression.

**COMPETENCY IN MEDICAL KNOWLEDGE 2:** Septal reduction therapy provides a targeted intervention that addresses the chronic effects of afterload on cardiac energy dynamics, restoring mechanical efficiency by reversing the Anrep effect. This study introduces the Anrep Triad—elevated afterload, increased contractility, and prolonged systolic duration—as a hemodynamic marker that can assist in the clinical evaluation of HOCM. By integrating this triad into routine diagnostic assessments, clinicians can better anticipate disease trajectory, personalize treatment strategies, and ultimately improve patient prognosis and quality of care. The suppression of the Anrep effect posttherapy offers not only symptomatic relief but also long-term improvement in cardiac function.

**TRANSLATIONAL OUTLOOK:** The implications of this study extend beyond HOCM, encouraging further research into the chronic activation of the Anrep effect in other cardiovascular conditions characterized by long-term high afterload, such as aortic stenosis, chronic hypertension, and heart failure with preserved ejection fraction. Investigating the molecular and physiological pathways of the Anrep effect in these settings can lead to the development of novel therapeutic strategies aimed at reducing the heart's mechanical and energetic burden, ultimately resulting in better outcomes for a wide range of patients with elevated afterload.

## Funding support and author disclosures

Dr Maack is funded by the Deutsche Forschungsgemeinschaft (DFG; SFB-1525/project No. 453989101 and Ma: 2528/8-1); and is an advisory board member for Bristol Myers Squibb, Boehringer Ingelheim, AstraZeneca, Servier, Amgen, Novo Nordisk, Bayer, Novartis, Edwards, and Berlin Chemie. Dr Sequeira is supported by a research fund from Bristol Myers Squibb and the Deutsche Forschungsgemeinschaft (DFG; No. 530849567); and has received research funding from Bristol Myers Squibb. All other authors have reported that they have no relationships relevant to the contents of this paper to disclose.
